# Puerperal septic shock complicated with symmetrical peripheral gangrene: A case report

**DOI:** 10.1097/MD.0000000000037571

**Published:** 2024-03-29

**Authors:** Yue Wang, Cen Tang, Yajin Li, Wanqin Hu

**Affiliations:** a Department of Obstetrics, The Second Affiliated Hospital of Kunming Medical University, Kunming, China.

**Keywords:** gangrene, puerperal sepsis, septic shock, symmetrical peripheral gangrene

## Abstract

**Rationale:**

Puerperal sepsis is a life-threatening condition caused by infection that can rapidly progress to multisystem infection and toxin-mediated shock. Symmetrical peripheral gangrene is defined as symmetrical distal ischemic damage in two or more sites in the absence of major vascular occlusive disease. The syndrome is devastating and rare. In this study, we introduce a case of puerperal septicemia complicated by symmetrical peripheral gangrene.

**Patient concerns:**

A 23-year-old woman delivered a live female infant vaginally after cervical balloon dilatation at 39 weeks of gestation. Persistent hyperthermia developed on the first postpartum day. After experiencing ventricular fibrillation, acute liver failure, and acute pulmonary edema, she developed blackened extremities on the 5th postpartum day.

**Diagnoses:**

Puerperal septicemia complicated by symmetrical peripheral gangrene.

**Interventions:**

Upon transfer to our hospital, the patient was enrolled in the intensive care unit and underwent anti-infective and amputation surgery.

**Outcomes:**

After the surgery, the patient recovered well and was successfully discharged from the hospital.

**Lessons:**

Early detection and timely treatment is the best way to reduce the mortality and sequelae of puerperal sepsis. Physicians should be alert to the possibility of comorbid symmetrical peripheral gangrene when sepsis patients present with hepatic impairment.

## 1. Introduction

Puerperal sepsis is a life-threatening disease and one of the major causes of maternal mortality, with a prevalence of 0.002% to 0.04% of all deliveries.^[1]^ Currently, the overall maternal mortality rate is decreasing, but there is a trend towards an increase in the mortality rate due to sepsis.^[2]^ Puerperal sepsis is defined as infection-induced organ dysfunction that may occur in the postpartum period. It is a systemic inflammatory response caused by infection and can be classified into sepsis, severe sepsis, and septic shock according to its severity. In a retrospective analysis of 74 intensive care unit (ICU) inpatients, systemic inflammatory response syndrome incidence was 59%, severe sepsis 24%, and septic shock 3%.^[3]^ From initial recognition of sepsis to progression to severe sepsis and shock, it is a rapid process; therefore, early diagnosis and immediate initiation of appropriate treatment are critical.

symmetrical peripheral gangrene (SPG) is a rare clinical condition with acute attacks on or above the extremities and without obstruction or vasculitis of the supplying arteries.^[4]^ It may manifest unpredictably in conditions associated with sepsis, low output states, vasospasm, myeloproliferative disorders, or hyperviscosity syndromes. The disease has a high mortality rate, and survivors often have multiple limb amputations.^[5]^ The incidence of postpartum septic shock complicated with SPG is extremely low and has not been reported worldwide. Here, we report a young mother who experienced puerperal sepsis after delivery that progressed to septic shock and SPG.

## 2. Case report

A 23-year-old woman was transferred to our hospital with shock on postpartum day 18. Eighteen days ago, she delivered a live baby girl vaginally after induction of labor by cervical balloon dilatation in a local hospital with postpartum hemorrhage of 400 mL. The patient developed recurrent fever on the first postpartum day, with a maximum temperature of 41.7°C. On postpartum day 2, ventricular fibrillation occurred, followed by acute hepatic and renal failure and acute pulmonary edema. She was transferred to the ICU and stabilized slightly after active treatment. On postpartum day 5, her distal extremities are bruised. On postpartum day 18, the patient was transferred to our hospital in critical condition, with apathy, obvious swelling of the limbs, and blackened gangrene with blisters breaking out at the extremities. The patient was previously healthy, with no internal medicine-related diseases, no immune system-related diseases, denied a history of trauma, claudication, arthralgia, no history of smoking, alcohol consumption, and no family history of major diseases.

**Figure 1.**
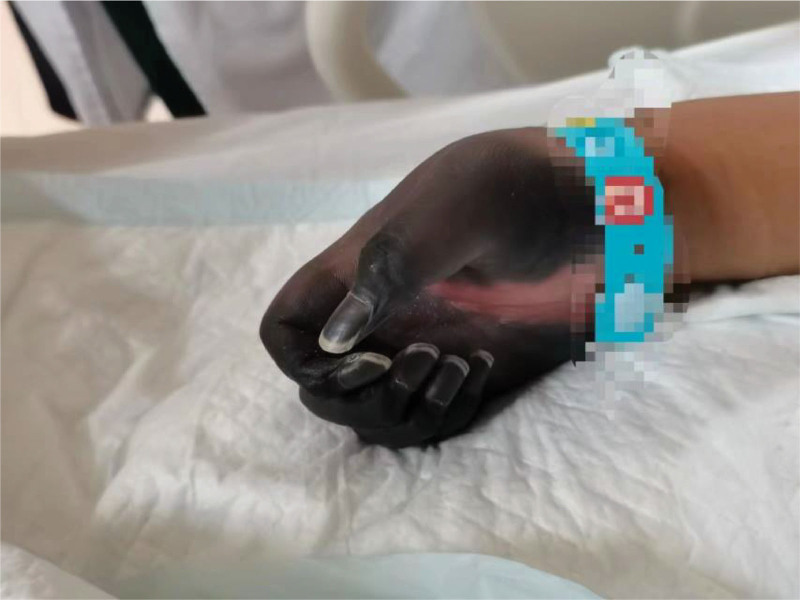
Gangrene in both upper limbs.

**Figure 2.**
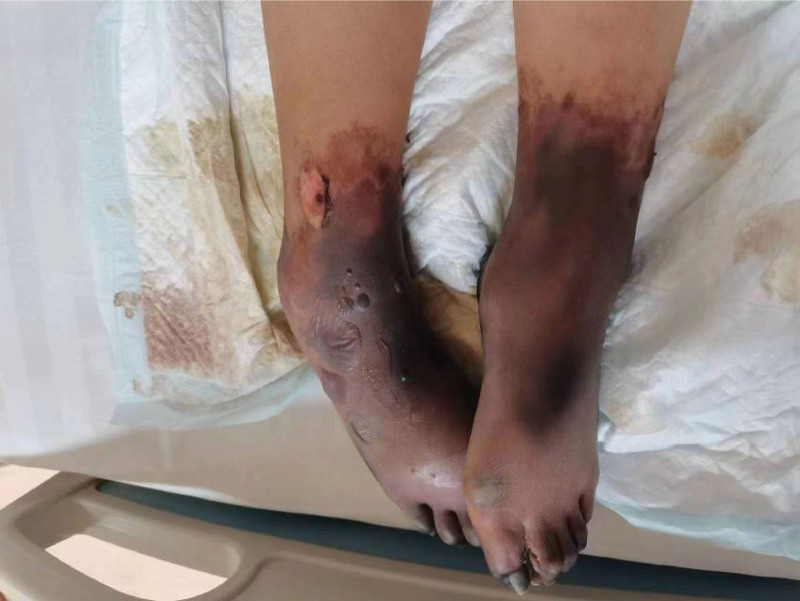
Gangrene with scattered blisters on both lower limbs.

On physical examination, her body temperature was 38.4°C, heart rate was 112 beats/min, blood pressure was 107/80 mm Hg, respiration rate was 33 beats/min, and oxygen saturation was 98% (oxygen 6 L/min by mask). She was in poor general condition, apathetic, and had sluggish pupillary reflexes to light. She was unable to complete repartee and command movements. Extremities were gangrenous, with high muscle tension, no retraction of the limbs under pain stimulation, and scattered blisters were seen on both lower limbs (Figs. 1 and 2). Specialized examination: a small amount of bloody vaginal discharge, no detectable odor, the uterus was located one transverse finger above the pubic symphysis, and there was no obvious active vaginal bleeding. Laboratory results: leukocytes 11.21 × 109/L, neutrophils 90.90%, hemoglobin 103 g/L, platelets 53 × 109/L, prothrombin time (PT) 16.8 s, activated partial thromboplastin time (APTT) 38.0 s, D-dimer 13.82 ng/mL, K 3.48 mmol/L, albumin 24.9 g/L, hydrogen ion concentration (PH) 7.43, lactic acid 2.5 mmol/L, brain natriuretic peptide (BNP) 525.3 ng/L, calcitonin 0.850 ng/L, blood amylase 233 U/L, urine amylase 837 U/L. She had a Sequential Organ Failure Assessment (SOFA) score of 7 on admission.

### 2.1. Diagnosis and treatment

**Figure 3.**
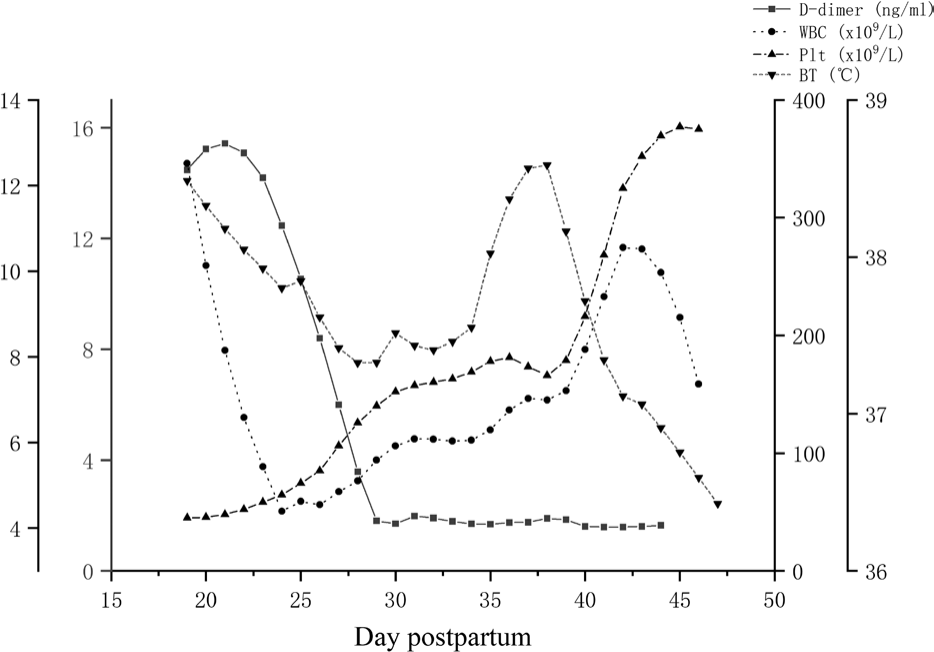
Changes in the patient’s temperature and laboratory test indexes on postpartum day 19–47.

The patient was immediately transferred to the ICU for shock, and given empiric anti-infection, fibrinogen supplement, levofloxacin, rehydration, anti-infection, anticoagulation, acid suppression, liver preservation, expectoration, and other supportive treatment. The condition deteriorated on postpartum day 20 (the second day of admission to our hospital). The body temperature continued to rise to 38.7°C; scattered blood spots and bruises all over the body; coagulation function indexes continued to be higher or lower than the normal range, and low molecular heparin anticoagulation treatment was given after evaluation. Blood and urine cultures detected Pseudomonas tropicalis; sputum culture detected Corynebacterium striatum, and culture of traumatic secretions detected Staphylococcus mansoni subspecies; according to the results of culture and drug sensitivity, the antibiotics were adjusted to vancomycin + imipenem + caspofungin + amphotericin B. After multidisciplinary consultation, considering the severity of SPG necrosis in the patient’s extremities and the severity of systemic infection symptoms, amputation surgery should be performed as soon as possible. With effective treatment over some time, the patient’s consciousness was restored, and the systemic infection was reduced compared to before. Therefore, bilateral lower limbs were amputated on postpartum day 23 and bilateral forearms were amputated on postpartum day 30. The patient recovered well after surgery and her vital signs were stable, so she was transferred to the general obstetrics ward on postpartum day 44. The blood culture was negative, urine and sputum cultures still detected Klebsiella pneumonia and imipenem anti-infective treatment was continued. On postpartum day 47, the patient’s condition improved; the highest body temperature in 24 hours was 36.5°C. Both forearms and feet had been amputated, with normal sensation in the stumps, normal blood circulation, and good muscle strength, and was discharged after the doctor’s evaluation (Fig. 3 and Figure S1, Supplemental Digital Content, http://links.lww.com/MD/L967. Changes in the patient’s temperature and laboratory test indexes on postpartum day 19–47). After discharge, the patient continued to receive imipenem anti-infective treatment at the local hospital for one week. Two consecutive blood and urine cultures were negative, and the prognosis was good.

## 3. Discussion

Puerperal sepsis is one of the most important causes of maternal mortality during pregnancy, with a prevalence of 0.002% to 0.04% of all deliveries.^[1]^ In recent years, the overall maternal mortality rate has decreased, but the maternal mortality rate for complicated sepsis has increased, accounting for 11.4% to 23% of maternal deaths.^[2]^ Normal postnatal physiologic changes during pregnancy may mask early signs of sepsis, often delaying the diagnosis of sepsis and increasing maternal mortality.^[6]^ The main sources of puerperal sepsis infection are intrauterine infections, wound infections, urinary tract infections, and pneumonia, and a definitive source of infection could not be identified in approximately 30% of cases.^[7]^ The most common pathogens involved are Escherichia coli, group A and B streptococci, Staphylococcus aureus, etc.; about 15% are mixed infections with multiple pathogens.^[8]^ A study of maternal mortality and morbidity in the UK and Ireland from 2009 to 2012 reported that 25% of maternal deaths were due to sepsis, and 63% of maternal deaths due to sepsis were due to informal care and failure to recognize, diagnose, and manage the disease promptly.^[9]^ Early clinical diagnosis of puerperal sepsis is based on a range of signs and symptoms. It relies primarily on the physician’s clinical experience rather than objective laboratory indicators for early recognition. Recognizing and intervening in pregnancy and puerperal sepsis is an important measure to reduce maternal mortality.^[10]^

This patient had a rare case of puerperal septic shock. She sustained an elevated temperature after delivery. However, the failure of the local hospital to provide early recognition and timely therapeutic interventions resulted in poor therapeutic outcomes, and septic shock and gangrene of the extremities had occurred by the time she was transferred to our hospital. After admission to our hospital, she was immediately treated aggressively and effectively. The results of blood, urine, sputum, and wound secretion culture suggested that the patient was a rare mixed infection of Pseudomyces tropicalis, Corynebacterium striatum, and Staphylococcus mansoni subspecies. Because the patient was transferred to our hospital from another hospital on the 18th day postpartum, it was difficult to determine where the initial source of the patient’s infection came from. After adjusting antibiotics based on culture and drug sensitivity results, the patient’s condition was under control. However, the dry gangrenous condition of his limbs was irreversible. To avoid aggravation of necrosis and complications of infection, the patient underwent amputation of both forearms and feet. After the operation, the patient recovered well and was discharged from the hospital.

There are no reports of SPG as a sequela of puerperal septic shock. The exact pathogenesis of SPG is unknown, but most SPG is associated with using vasopressor drugs in patients with disseminated intravascular coagulation (DIC). The distal ischemic changes of SPG in the patient reported in this case may have been due to the combination of a hyperemic state with microvascular occlusion in DIC, and the use of vasoconstrictor drugs administered early in the course of shock exacerbated these ischemic changes.^[11]^ SPG is characterized by symmetrical skin and distal necrosis followed by gangrene in two or more distal sites without obstruction of the aorta. There is a characteristic SPG triad: shock, DIC, and natural anticoagulant depletion (protein C, antithrombin). In recent years, risk factors for natural anticoagulant depletion have been identified, most notably acute ischemic hepatitis (“shock liver”), which occurs in at least 90% of patients with SPG. Moreover, there is a characteristic time interval (2–5 days, median 3 days) between the onset of shock/shock liver and the beginning of ischemic injury secondary to peripheral microthrombosis, reflecting the time required to develop severe depletion in hepatically-synthesized natural anticoagulants.^[12]^ This suggests that failure of the protein C and antithrombin natural anticoagulant systems due to insufficient hepatic synthesis of these crucial proteins profoundly disturbed procoagulant–anticoagulant balance could explain the microvascular thrombosis and associated limb loss.^[13]^ SPG has been reported to have a mortality rate of approximately 18% to 40%, with a high frequency of multiple limb amputations in survivors.^[5]^ The Early stages of SPG are not amputated because secondary infection of the necrotic tissue is uncommon. Still, the pre-gangrenous develops distinct borders over time and becomes outright gangrene.^[14]^ So, identifying and treating the potential cause can help to stop and prevent further progression of SPG. Notably, this patient developed cardiogenic shock and acute hepatic failure on postpartum day 2, followed by bruising of the distal extremities three days later, which is consistent with a “shock liver.” Since shock liver precedes the onset of limb ischemia by several days, it warns us to be on the lookout for SPG.

In summary, early detection and prompt treatment is the best way to reduce mortality and sequelae of puerperal sepsis.^[10]^ “A hospital considering a 1-hour bundle for management of sepsis” should be given immediately to a pregnant woman with suspected sepsis, including fluid resuscitation, correction of hypoxia, initiation of empiric therapy with antimicrobials, and narrowing and focusing antibiotic coverage once culture results are obtained.^[15]^ In addition, clinicians should be aware that puerperal septic shock can be complicated with SPG, especially when shock liver is observed, and that therapeutic interventions are needed to preempt the onset of limb ischemia.

## Author contributions

**Formal analysis:** Yue Wang, Cen Tang, Yajin Li.

**Investigation:** Yue Wang.

**Project administration:** Yue Wang, Wanqin Hu.

**Writing – original draft:** Yue Wang.

**Writing – review & editing:** Wanqin Hu.

## Supplementary Material


