# Supplementary material for: Puerperal septic shock complicated with symmetrical peripheral gangrene: A case report
Source: Medicine (Baltimore). 2024 Mar 29;103(13):e37571. doi: 10.1097/MD.0000000000037571 (PMC10977569; doi:10.1097/MD.0000000000037571)
Supplement: Supplementary file 1 [file medi-103-e37571-s001.docx]

| **Parameter** | **Body temperature,℃** | **Lac**  **(0.5-2.2)** | **PLT, x10^9^/L**  **(125-130)** | **WBC, x10^9^/L**  **(3.5-9.5)** | **D-dimer**  **ng/ml**  **(0-0.05)** | **Pct ng/ml**  **(0.02-0.05)** |
| --- | --- | --- | --- | --- | --- | --- |
| **Day19** | 38.4 | 2.5 | 40 | 11.21 | 13.82 | 0.850 |
| **Day 20** | 38.7 | 1.8 | 47 | 12.99 | 17.05 |  |
| **Day 21** | 38.0 | 2.2 | 50 | 7.01 | 15.83 |  |
| **Day 22** | 37.7 | 1.7 | 65 | 6.62 | 13.6 |  |
| **Day 23** | 37.9 | 2.6 | 57 | 4.08 | 11.84 | 0.5 |
| **Day 24** | 38 | 2.1 | 56 | 4.78 | 12.6 | 0.3 |
| **Day 25** | 38 | 2.3 | 74 | 5.31 | 14.19 | 0.15 |
| **Day 26** | 37.5 | 1.9 | 87 | 4.71 | 8.71 | 0.6 |
| **Day 27** | 37.5 | 1.5 | 107 | 4.51 | 3.64 | 0.5 |
| **Day 28** | 37.4 | 2.3 | 123 | 4.99 | 2.6 | 0.61 |
| **Day 29** | 36.7 | 1.3 | 135 | 5.16 | 2.4 | 0.7 |
| **Day 30** | 37.8 | 1.7 | 170 | 6.16 | 2.05 | 0.34 |
| **Day 31** | 37.7 | 2.6 | 153 | 6.93 | 2.08 |  |
| **Day 32** | 37.6 | 2.6 | 158 | 6.34 | 1.7 | 0.44 |
| **Day 33** | 37.6 | 1.5 | 163 | 5.01 | 2.21 | 0.04 |
| **Day 34** | 36.7 | 2 | 160 | 5.92 | 1.64 | 0.03 |
| **Day 35** | 37.9 | 1.4 | 176 | 6.39 | 1.48 | 0.04 |
| **Day 36** | 38.5 | 1.2 | 189 | 6.85 | 1.64 | 0.04 |
| **Day 37** | 39.2 | 1.3 | 196 | 7.51 |  | 0.04 |
| **Day 38** | 39 | 1.7 | 169 | 7.34 | 2.09 | 2.79 |
| **Day 39** | 37.6 | 1.2 |  |  |  |  |
| **Day 40** | 37.5 | 2.1 | 166 | 6.97 |  | 1.61 |
| **Day 41** | 37.3 | 1.9 |  |  |  |  |
| **Day 42** | 37.2 | 1.5 | 370 | 10.9 | 1.04 | 0.38 |
| **Day 43** | 37.2 | 0.6 | 399 | 12.88 | 0.86 |  |
| **Day 44** | 37.0 | 1.3 | 384 | 10.83 | 2.17 |  |
| **Day 45** | 36.5 |  |  |  |  |  |
| **Day 46** | 36.6 |  | 355 | 6.39 |  |  |
| **Day 47** | 36.5 |  |  |  |  |  |
